# Vascular Development and Regeneration in the Mammalian Heart

**DOI:** 10.3390/jcdd3020023

**Published:** 2016-06-16

**Authors:** Oscar M. Leung, Bin Zhou, Kathy O. Lui

**Affiliations:** 1 Department of Chemical Pathology, The Chinese University of Hong Kong, Prince of Wales Hospital, Hong Kong, China; oscar5292003@yahoo.com.hk; 2 Li Ka Shing Institute of Health Sciences, The Chinese University of Hong Kong, Prince of Wales Hospital, Hong Kong, China; 3 Key Laboratory of Nutrition and Metabolism, Institute for Nutritional Sciences, Shanghai Institutes for Biological Sciences, Chinese Academy of Sciences, Shanghai 200031, China; zhoubin@sibs.ac.cn; 4 Institute of Neuroscience, State Key Laboratory of Neuroscience, CAS Center for Excellence in Brain Science and Intelligence Technology, Shanghai Institutes for Biological Sciences, Chinese Academy of Sciences, Shanghai 200031, China; 5 School of Life Science and Technology, ShanghaiTech University, Shanghai 201210, China

**Keywords:** vascular development, vascular endothelial cells, angiocrine factors, heart regeneration

## Abstract

Cardiovascular diseases including coronary artery disease are the leading cause of death worldwide. Unraveling the developmental origin of coronary vessels could offer important therapeutic implications for treatment of cardiovascular diseases. The recent identification of the endocardial source of coronary vessels reveals a heterogeneous origin of coronary arteries in the adult heart. In this review, we will highlight recent advances in finding the sources of coronary vessels in the mammalian heart from lineage-tracing models as well as differentiation studies using pluripotent stem cells. Moreover, we will also discuss how we induce neovascularization in the damaged heart through transient yet highly efficient expression of VEGF-modified mRNAs as a potentially therapeutic delivery platform.

## 1. Introduction

Recently, it has been reported that heart vessels not only supply nutrients and oxygen to the heart, but also provide regenerative cues in pathological conditions such as coronary artery disease and subsequently myocardial infarction (for review, see [1,2]). Every year, approximately 17.5 million people die from cardiovascular diseases, the leading cause of mortality, accounting for 31% of all deaths worldwide [3]. Unfortunately, the heart muscle does not significantly regenerate, and disease progression from blocked heart vessels in some patients cannot be delayed by medical or surgical interventions, including balloon angioplasty or coronary artery bypass [3]. Therefore, the regeneration of coronary arteries through stem-cell-based tissue engineering, paracrine-factor-based approaches, or both offers great potential in the treatment of cardiovascular diseases. Moreover, regenerative induction that recapitulates normal coronary vessel development is more direct and desirable. Together, understanding the developmental origins of coronary vessels in the heart could shed light on novel treatment options for both congenital and adult cardiovascular diseases.

There are three basic tissue layers that form a coronary artery including the endothelial cell, smooth muscle cell, and fibroblast layers. In fact, the endothelium is the first layer of cells formed during coronary artery development, and the first site where coronary artery disease begins in adults. Therefore, identifying the cellular origins and sources of coronary vessels is essential in tracing development as well as uncovering mechanisms of coronary artery disease and regeneration. In this review, we will highlight recent advances in finding the origins and sources of coronary vessels in the heart using lineage-tracing mouse models and pluripotent stem cell tools. Additionally, we will also discuss a potential therapeutic delivery platform via the transient yet highly efficient expression of modified mRNAs for upregulating paracrine factors of interest and inducing neovascularization in the damaged heart.

## 2. The Origin of the Vascular System in the Mammalian Heart

By virtue of the Cre/loxp technology, we are now able to perform fate mapping experiments and specifically trace the origins of the vascular system of the developing heart. Using the Islet1 (Isl1)-Cre lineage tracing mouse model, the multipotent cardiovascular progenitors of the second heart field, the Isl1^+^ progenitors, have been found to give rise to vascular smooth muscle and endothelial cells that form most of the outflow tract region of the developing heart [4]. More recently, using the Nkx2-5-Cre lineage tracing zebrafish and mouse models, it has also been reported that the Nkx2-5^+^ progenitors give rise to endothelial cells of the pharyngeal arch arteries that form the great vessels of the developing heart [5]. Nevertheless, the first heart field progenitors, particularly the Hcn4^+^ progenitors, are found to give rise to cardiomyocytes only, but neither the vascular smooth muscle nor the endothelial cells of the developing heart [6]. Furthermore, the WT1^+^ [7] epicardial progenitors contribute to the majority of vascular smooth muscle and a small number of endothelial cells [7,8], and Tbx18^+^ [9] epicardial progenitors contribute to vascular smooth muscle cells, but not to vascular endothelial cells of the developing heart. Intriguingly, cardiovascular progenitors that give rise to great vessels or endocardium of the heart do not seem to make a significant contribution to the development of coronary vessels, as demonstrated in Isl1-Cre, Nkx2.5-Cre, WT1-Cre, and Tbx18-Cre mice [10,11]. Specifically, although 50% of the endocardium is labeled by Isl1-Cre, only 3% of the coronary vessels in the outer layer of ventricle wall are derived from the Isl1^+^ lineage [10]. Altogether, the coronary vessels could have different origins from the great vessels and endocardium of the mammalian heart.

It was once thought that the coronary vessels were derived by angiogenic sprouting from pre-existing embryonic vasculatures in the developing heart. Recently, this concept has been challenged by several reports documenting the (pro)epicardium [7,9], the sinus venosus [12], and the endocardium [10,13,14] as the major origins of coronary vessels. It is less clear whether (pro)epicardial cells make any significant contribution to coronary endothelial cells, although a subset of proepicardial cells is found to generate up to 17% of the coronary endothelial cells in the developing heart [15,16]. In addition, more recent studies show that the proepicardium is not the primary source of coronary endothelial cells, but of coronary smooth muscle cells, as demonstrated by lineage tracing experiments using WT1-Cre [7] and Tbx18-Cre [9] mice. A recent study using a G2-Gata4-Cre transgene also indicates that at least 20% of the embryonic coronary arterial and capillary endothelial cells are derived from the septum transversum/proepicardial endothelial compartment [17]. To determine the origin of coronary endothelial cells, histological analysis, clonal assays and cardiac organ culture in mice have revealed the sinus venosus as a common origin of the endothelium of coronary arteries and veins [12]. Coronary vessels arise from angiogenic sprouting from sinus venosus where blood returns to the embryonic heart: the sprouting venous endothelial cells dedifferentiate as they migrate and invade the myocardium, the invading cells differentiate into arteries, and capillaries with cells on the vessel wall redifferentiate into veins again [12]. These results show that some differentiated venous cells remain plastic during development; and cardiac signals can direct them to dedifferentiate/redifferentiate into coronary arteries, capillaries, and veins [12,18].

## 3. Subepicardial Endothelial Cells Are One of the Major Sources of Coronary Vessels

Recently, the endocardium has been found to be the primary origin of coronary endothelial cells [10,13,14]. Wu *et al.* have reported that ventricular endocardial cells generate coronary arteries by angiogenesis using the Nfatc1-Cre lineage tracing model where Nfatc1 only marks the endocardium but not endothelial cells of coronary vessels [14]. Upon transplantation of the Nfatc1-Cre^eGFP^ embryonic ventricle into VEGF_120_-containing matrigel, the Nfatc1 precursor cells are found to migrate and sprout through the ventricular wall to form endothelial tubes [14]. Moreover, the myocardial VEGF_A_ to endocardial VEGFR2 signaling axis is required for endocardial cells to differentiate into coronary endothelium as no coronary arteries are found in E14.5 Nfatc1-Cre;;Vegfr2^fl/fl^ embryos following endocardium-specific deletion of VEGFR2 [14]. Intriguingly, coronary veins can be found in these embryos. In contrast to Red-Horse *et al.* who propose a common origin for coronary arteries and veins [12], Wu *et al.* found that coronary arteries and veins could have a different origin, as the endocardium is less likely to generate coronary veins [14].

While Wu *et al.* show that ventricular endocardium is the primary source of intramyocardial coronary vessels using the Nfatc1-Cre lineage-tracing line [14], Tian *et al.* demonstrate that only little ventricular endocardial cells migrate into the myocardium of free ventricular wall in the embryonic heart [10]. Using the Apelin-1-Cre lineage tracing line, it was found that the Apelin-1-derived subepicardial endothelial cells are the major source of intramyocardial coronary vessels in the embryonic heart (E10.5–11.5) which invade the compact myocardium to form coronary arteries and remain on the surface to produce the vein [10]. Therefore, the coronary arteries and veins have a common origin in the developing heart. One caveat to the inconsistent observation is that Nfatc1-Cre not only labels endocardial cells of the ventricles but also a subset of endocardial cells of atria and sinus venosus. Therefore, it remains possible that coronary vessels are derived from endocardial cells of the atria and sinus venosus in addition to those from the ventricles [10,13,19]. Indeed, Tian *et al.* confirm that Apelin-Cre labels the first coronary vascular population derived from sub-epicardial endothelial cells following tamoxifen induction at E10.5, which ultimately gives rise to vascular endothelial cells of the outer myocardial wall of neonatal hearts [13]. Nfatc1-Cre labels the second coronary vascular population, which is located in the interventricular septum and inner myocardial wall at later embryonic and early neonatal stages [13]. By using the natriuretic peptide receptor 3 (Npr3) lineage-tracing line, which labels ventricular endocardium but not the sinus venosus, Zhang *et al.* showed that Npr3-CreER labeled endocardium minimally contributed to the coronary endothelium in the embryonic ventricular free wall [19]. Therefore, the ventricular endocardium gives rise to some coronary vessels, but the majority of coronary vessels are derived from Apelin-Cre-labeled subepicardial endothelial cells that give rise to endocardial cells of the sinus venosus and atria. The difference in conclusions in the origins of coronary arteries may be attributed to the difficulty in finding specific promoters that are expressed by the target cell population of interest. For instance, Nfatc1 is reported to be expressed in coronary endothelial cells of the developing heart [20]. Nfatc1-Cre also labels the endocardium that contributes to cardiac fat [21]. Moreover, although the Apln-Cre lineage tracing line could trace the cell fate of subepicardial endothelial cells at later stages, the tool alone could not define the origins of these subepicardial endothelial cells that form the majority of coronary vessels. More advanced technologies are, therefore, needed to identify and accurately quantify the different origins of coronary vessels.

## 4. Understanding Vascular Development via Pluripotent Stem Cells

Recently, it has been reported that it is safe to transplant clinical-grade human embryonic stem cell (hESC)-derived cardiac progenitor cells into patients with severe heart failure [22]. Symptomatic improvement was also observed with evidence of new-onset contractility in the previously non-revascularized area [22]. Although more detailed analysis in functional efficacy is needed, pluripotent stem cells such as hESCs are instrumental for recapitulating heart development and for studying the possible origin of blood vessels both *in vitro* and *in vivo*. Using Cre-based lineage tracing technology and direct differentiation assays in ESC cultures, the blood vessel-forming cells including vascular smooth muscle cells and endothelial cells are found to be derived from various cardiovascular progenitors, including Mesp1^+^ [23,24], Isl1^+^ [4,25,26], and c-Kit^+^Sca-1^+^ [27] cells, to name a few. 

Specifically, Mesp1 is one of the several transcription factors that have been identified to be essential in heart development [28]. Mesp1 is found to be the earliest marker with expression detected at embryonic day 6.5 (E6.5) along the primitive streak. This marks the mesodermal population that gives rise to both the primary and secondary heart fields [29]. Importantly, severe cardiac defects are observed in Mesp1-null embryos which usually die at E10.5 [30]. Using the dual cardiac fluorescent reporter MESP1(mCherry/w)NKX2-5(eGFP/w) line in the hESC system, one observes the pre-cardiac MESP1^+^ mesoderm and their further commitment towards the cardiac lineage with activation of the NKX2-5^+^ cardiovascular progenitors [31]. In addition, using the ISL1-Cre lineage tracing human ESC line, one observes commitment of the second heart field cardiovascular progenitors to cardiomyocytes and vascular smooth muscle and endothelial cells upon direct differentiation *in vitro* and *in vivo* [25,26]. Importantly, the Isl1 progenitors mainly contribute to vascular smooth muscle and endothelial cells of the outflow tract and endocardium of a developing Isl1-Cre mouse heart [4]; moreover, we also observe co-localization of ISL1^+^CD31^+^ cells near the outflow tract region of a human fetal heart [25,26], indicating the possible commitment of Isl1^+^ cardiovascular progenitors into large blood vessels of the heart. Nevertheless, though it remains controversial whether the c-KIT^+^ progenitors contribute to cardiomyocytes [32,33,34], one observes commitment of the c-Kit^+^Sca-1^+^ progenitors to vascular endothelial cells and smooth muscle cells both *in vitro* and *in vivo* by purifying and differentiating c-KIT^+^ cells from ESCs [27].

## 5. Driving Vascular Regeneration in the Damaged Heart via Acquired Angiogenesis

Recently, there have been a number of clinical trials in transplanting cardiac stem cells such as c-kit^+^ cells [35], cardiosphere-derived cells [36], as well as mesenchymal stem cells [37,38] into the damaged myocardium with the hope of improving heart function after cell transplantation. In these trials, direct differentiation of the transplanted cells into functional myocardial cells was not observed. Instead, the main mechanism by which the transplanted cells improve heart function, if any, is believed to mediate through the secretion of paracrine factors that are often angiogenic factors (for review, please see [1,2,39]) that promote a number of repair responses including acquired neovascularization, increased cell replication, reduced cell death, inhibited hypertrophy, and altered extracellular matrix deposition. Together, improved blood perfusion, reduced fibrosis, and enhanced left ventricular function are observed in the ischemic heart treated with transplanted cells. Therefore, understanding how to acquire local neovascularization and amplify the angiogenic signals in the injured myocardium might provide an alternative strategy for promoting heart regeneration following ischemic heart diseases.

A number of angiogenic factors have been proposed to induce neovascularization following ischemic injury, such as hepatocyte growth factor/HGF [36], insulin-like growth factor-1/IGF-1 [36], stromal cell-derived factor-1/SDF-1 [40], thymosin beta 4 [41], and vascular endothelial growth factor/VEGF [26,42]. By virtue of the epicardial WT1-CreER lineage tracing line, one would find that there is an increase in the adult endogenous WT1-derived cells following myocardial infarction, but such a slight increase is insufficient to improve heart function after injury; moreover, the WT1-derived cells are mostly cardiac fibroblasts [7,42]. Importantly, local delivery of angiogenic thymosin beta 4 [41] or VEGF [42] following myocardial infarction not only induced angiogenesis and improved left ventricular ejection fraction of the ischemic heart, but also activated the quiescent adult epicardial progenitor cells to further differentiate into *de novo* cardiomyocytes in the injured myocardium. This could avoid the caveats of limited graft survival and immune rejection (for review, see [43,44]) following transplantation of non-self progenitor cells derived from human pluripotent stem cells that could, in theory, contribute to new cardiac muscle and replace the damaged myocardial cells following ischemic injury.

Nonetheless, there are possible complications associated with angiogenic factor-based therapy for ischemic heart diseases such as limited half-life of the protein, or genomic integration and risk of mutagenesis as a result of viral vector expression. Moreover, prolonged expression of the angiogenic factor via DNA plasmids could also introduce unwanted side effects such as edema as a result of prolonged exposure to VEGF [42]. Indeed, various clinical trials with an attempt to induce revascularization in the ischemic heart by introducing VEGF recombinant protein, naked cDNA, non-viral plasmid, or adenoviral plasmid were largely disappointing (for review, see [2]). Recently, VEGF-containing biodegradable scaffolds such as hydrogel [45], collagen [46,47], or self-assembling peptide nanofibers (NFs) [48] have been implanted to increase the retention of VEGF in the infarcted heart, which showed better improvement in revascularization and left ventricular function in the ischemic heart. However, more studies are underway to determine the long-term efficacy and safety such as fibrosis and immune rejection against the implanted scaffolds. More recently, we [26,42] have utilized VEGF-modified mRNAs as a tool for local, transient, and non-immunogenic expression of VEGF in the ischemic myocardium following myocardial infarction. We demonstrated a highly efficient VEGF protein expression via modified mRNA in a dose- and time-dependent manner, inducing local revascularization with improved left ventricular function. Therefore, our results showed that angiogenic factors such as VEGF in the form of modified mRNA offer therapeutic potential in driving heart regeneration following ischemic injury. Nevertheless, more studies are needed to prove the safety and efficacy of using angiogenic factor-based modified mRNA in large animal and primate studies.

## 6. Future Perspectives

Unraveling the origin of coronary vessels could shed light on development of novel therapeutics for treating ischemic heart diseases, which often lead to end-stage heart failure. Moreover, the generation of new blood vessels in the injured heart with an increased secretion of endothelial cell-derived growth factors, namely angiocrine factors, via transplantation of human pluripotent stem-cell-derived endothelial progenitor cells or acquired expression of angiogenic factors could also promote heart regeneration (for review, see [1,2]). In fact, one of the main mechanisms by which the transplanted “stem” cells such as c-kit^+^ cells or mesenchymal stem cells repair a damaged heart is mediated through the secretion of paracrine factors (for review, see [2]). In addition, regeneration through induction of organ-specific angiocrine factors has also been reported in the liver [49], lung [50], bone [51], and skeletal muscle [52]. Therefore, the identification of novel organ-specific self-repairing angiocrine factors promotes regeneration not only of the heart but also, possibly, of multiple organ systems. Nevertheless, current protocols in inducing *de novo* differentiation of adult heart progenitor cells into cardiomyocytes of the damaged myocardium are inefficient; more investigation is needed to identify novel efficacious angiocrine factors in this regard. Moreover, more research is also needed to delineate the mechanisms by which angiocrine factors mediate heart regeneration, and to confirm the safety and efficiency of using novel delivery strategies such as modified mRNA to reintroduce regenerative angiocrine factors for heart repair.
